# “She is courageous because she does not care what people think about her…”: attitudes toward adolescent contraception use among Rwandan family planning providers and adult female modern contraceptive users

**DOI:** 10.1186/s12978-022-01517-4

**Published:** 2022-11-04

**Authors:** Hilary Schwandt, Angel Boulware, Julia Corey, Ana Herrera, Ethan Hudler, Claudette Imbabazi, Ilia King, Jessica Linus, Innocent Manzi, Madelyn Merritt, Lyn Mezier, Abigail Miller, Haley Morris, Dieudonne Musemakweli, Uwase Musekura, Divine Mutuyimana, Chimene Ntakarutimana, Nirali Patel, Adriana Scanteianu, Biganette-Evidente Shemeza, Gi’anna Sterling-Donaldson, Chantal Umutoni, Lyse Uwera, Madeleine Zeiler, Seth Feinberg

**Affiliations:** 1 Western Washington University, Bellingham, WA USA; 2 Spelman College, Atlanta, USA; 3 Wheaton College, Norton, USA; 4 Northwest Vista Community College, San Antonio, USA; 5 Whatcom Community College, Bellingham, USA; 6 INES, Ruhengeri, Rwanda; 7 Xavier University, New Orleans, USA; 8 University of Maryland Baltimore County, Baltimore, USA; 9 SUNY Oswego, Oswego, USA; 10 Western Oregon University, Monmouth, USA; 11 Eastern Oregon University, La Grande, USA; 12 University of Kentucky, Lexington, USA; 13 Arcadia University, Glenside, USA; 14 Rutgers University, New Brunswick, USA; 15 Drexel University, Philadelphia, USA

**Keywords:** Rwanda, Family planning, Adolescent, Contraception

## Abstract

**Introduction:**

In Rwanda, only 20% of sexually active unmarried young women use family planning as compared to 64% of married women. Adolescence is an important time of growth and development that often includes the initiation of sexual activity. Sexually active adolescents need support in accessing contraceptive services to prevent negative health outcomes. In sub-Saharan Africa, the adolescent population represents a large share of the total population and that proportion is predicted to expand over time. Adolescent contraceptive needs have largely been unmet, and with growing numbers, there is increased potential for negative health sequelae. Due to the low use of contraception by adolescents in Rwanda, and the growing population of adolescents, this study aims to explore the perspectives of family planning providers and adult modern contraceptive users on adolescent contraceptive use. Inclusion of adult community members in the study is a unique contribution, as research on adolescent contraceptive use in sub-Saharan Africa relies primarily on perspectives from adolescents and family planning providers.

**Methods:**

This qualitative study in 2018 utilized 32 in-depth interviews with modern contraceptive users and eight focus group discussions with family planning providers. Respondents were from Musanze and Nyamasheke districts in Rwanda, the districts with the highest and lowest modern contraceptive use among married women, respectively. Coding was conducted in Atlas.ti.

**Results:**

Stigma regarding premarital sex results in barriers to adolescent access to contraceptive services. Family planning providers do provide services to adolescents; however, they often recommend secondary abstinence, offer a limited method selection, and accentuate risks associated with sexual activity and contraceptive use. Providers support adolescent clients by emphasizing the need for privacy, confidentiality, and expedient services, particularly through youth corners, which are spaces within health facilities designed to meet youth needs specifically. Modern contraceptive-using adult female community members advocate for youth access to contraception, however mothers have mixed comfort discussing sexual health with their own youth.

**Conclusion:**

To destigmatize premarital sexual activity, government efforts to initiate communication about this topic must occur at national and community levels with the goal of continued conversation within the family. The government should also train family planning providers and all health personnel interacting with youth on adolescent-friendly health services. Dialogue between community members and family planning providers about adolescent access to contraceptive services could also reduce barriers for adolescents due to community members’ generally supportive views on adolescent contraceptive use. Efforts to engage adolescent caregivers in how to talk to youth about sex could also contribute to expanded use.

## Plain Language summary

In Rwanda, youth who are having sex use family planning less than married women. This study involved asking family planning providers and adults what they think about youth using family planning. Data for this study was collected in 2018, and included 32 interviews with adult family planning users and eight group discussions with family planning providers in two areas of Rwanda. The findings show that Rwandans believe youth should not have sex before they are married. Family planning providers do provide youth with services; however, they often push stopping sexual activity, offer a smaller selection of family planning methods, and exaggerate risks associated with sex and family planning use. Family planning providers support youth by honoring their need for privacy, keeping their secrets, and providing fast services so fewer people see them at the clinic. Providers like to help adolescents in youth corners, which are special spaces within health facilities just for youth. Importantly, adult women who use family planning want youth in their community to be able to use family planning, too. In order to respond to the issues raised, the Rwandan government can start conversations in villages and more broadly about the need for youth to have access to family planning. The government should also teach family planning providers and anyone who comes into contact with youth to offer helpful and friendly services. Setting up spaces for adults to talk with family planning providers about youth access to family planning could also contribute to fewer barriers to services for adolescents.

## Introduction

There are approximately 261 million women aged 15–19 living in developing nations, representing 16% of all women of reproductive age. This number will increase to 286 million by 2030. Africa is the youngest world region, with 41% of the population under 15 years of age [1]. About 21 million pregnancies occur each year in this age group, accounting for 11% of all births—95% of which are in developing nations. Half of these pregnancies are unplanned, and half of those end in abortion—often unsafely [2]. The leading cause of death globally for female adolescents aged 15–19 years old is maternal conditions [3].

Among this age group, 12% desire contraception, and 57% of those use a modern method [2]. Adolescent modern contraceptive use has doubled globally over the last 10 years. In Africa, modern method use has increased from 4 to 15% in the same time period [4]. Despite increases in adolescent contraceptive use, adolescent unmet need continues to be twice as high when compared to adult women [5] and higher among adolescents in sub-Saharan Africa as compared to other world regions [6].

Due to the persistent low uptake of contraceptive methods among adolescents in sub-Saharan Africa, researchers have examined this issue in various contexts from the perspectives of both adolescents and providers. Research in Ghana demonstrated that youth who had visited health facilities were more likely to use contraceptive services [7]; however, adolescents report barriers to accessing contraceptive services including judgmental providers, privacy, and confidentiality concerns [8–10].

Similar research in South Africa found that family planning providers felt adolescents were afraid to access contraceptive services due to judgmental providers and privacy concerns [11], and replication from other studies suggest these are valid concerns. For example, Zambian providers restricted access based on age [8]. Providers in Uganda feel that adolescents should not be having sex [12]; therefore, Ugandan providers can be judgmental and provide limited information, methods options, and privacy [13]. In Nigeria, providers exclude adolescents from their client base due to concerns about promoting premarital sexual activity [14], leading them to encourage abstinence instead. Providers were mixed with some feeling that provision of contraception to adolescents violates cultural norms about premarital sexual activity while others were open to providing adolescents contraception [15]. Notably, Rwandan providers highlight the importance of privacy and confidentiality, but also a need for more training and fewer barriers at the community level [16]. Less research has been conducted on community members themselves, despite barriers to adolescent access and use of family planning at the community level by community members listed as a common issue for adolescent access.

Rwanda’s family planning program is widely recognized as successful [17–25]. In Rwanda in 2020, 58% of married women used modern contraceptives. That same year, 17% of young women aged 15–19 reported being sexually active; however, only 18% of unmarried sexually active adolescents reported using modern methods. Use by method among these sexually active unmarried adolescents was as follows: 7% used implants, 5% male condoms, 4% injectables, and 2% pills [26]. The Rwandan Government has indicated that adolescent reproductive health is a national priority [27]. In light of the disparity in contraceptive use between married women and sexually active unmarried youth in Rwanda, this study aims to understand the perspectives of family planning providers and female adult modern contraceptive users on adolescent use of contraception. The first objective is to understand how family planning providers receive, counsel, and provide contraception to adolescent clients, and what barriers to adolescent access exist. The second objective is to determine how community members who use modern contraception view adolescents’ access to and use of contraception.

Most studies on adolescent access to and use of contraception focus either on the adolescent or provider perspectives. In addition, they include speculation about barriers to adolescent contraceptive use from their communities without including community members in the study. This study offers a unique contribution to the literature through its inclusion of adult modern contraceptive-using community members’ perspectives. The viewpoints of adult community members on adolescent access to and use of contraception has been absent in the literature, generally, and specifically in Rwanda, too [8, 28, 29]. Since the success of Rwanda’s family planning program is highly attributable to the shift in community norms and interpersonal discourse propelled by the government’s mobilization campaign [23], viewpoints of adult community members are critical in gauging and improving acceptance of adolescent contraceptive usage. Additionally, most research on family planning provider’s perspectives on adolescent contraceptive access and use report on providers as barriers to adolescent access. While similar findings result from this study, providers also highlight concrete ways in which they facilitate adolescent access to family planning services in the ways that mesh with adolescents’ desires [10, 29]**.**

## Methods

This qualitative study was conducted using a phenomenological approach and postpositivist paradigm in the Musanze and Nyamasheke districts of Rwanda over two distinct time periods in February and July of 2018. These districts were selected because they represented the areas of the country with the highest and lowest rates of modern contraceptive methods among married women, respectively, at the time of data collection. To further contextualize these two districts, 84% of Musanze households have health insurance coverage compared to 76% of Nyamasheke households. In Musanze, 75% of women of reproductive age listen to the radio weekly compared to 57% of women in Nyamasheke. The total fertility rate in Musanze is 3.5 and 5.0 in Nyamasheke [30].

The research team conducted focus group discussions with community health workers and family planning nurses in Musanze and Nyamasheke. Specifically, the team conducted four focus groups in Musanze and Nyamasheke (two with nurses and two with community health workers in each district). Family planning providers were recruited purposively via phone calls and face-to-face interactions between NGO staff and governmental hospital staff knowledgeable of all providers in the districts. A priori, focus group discussions were set to size 8 to 12 participants—in order to have enough participants to generate a discussion, but not too many that participants would not have space to share. Provider type was the only difference between the groups; therefore, junior and senior providers could have been in the same group. A total of 88 respondents spent between 90 and 150 min with two native Kinyarwanda speakers moderating, note-taking, and recording the focus group. Study participants, the moderator, and the note taker were the only people present for each focus group. The focus group discussions took place inside private rooms in university or hospital buildings. The topic guide included questions about barriers for clients, strengths and weaknesses with current provision of services, and a range of questions regarding respondent opinions and perspectives on their daily interactions and observations as family planning providers, including responses to hypothetical scenarios regarding sexual activity and unwanted pregnancies for married and unmarried women and adolescents in Rwanda.

These data are augmented with 32 in-depth interviews held the same year with purposefully selected female modern contraceptive users who were older than 18 years of age. These interviews were conducted by Kinyarwanda speakers with 16 respondents in Musanze and 16 in Nyamasheke during July of 2018, and lasted on average 43 min. Sixteen respondents were selected, as originally the study design included eight current modern contraceptive users and eight recent modern method discontinuers. After study recruitment had commenced, confusion around the discontinuation study selection parameter arose, and the team realized participants were not selected by that study criterion. Modern contraceptive users were recruited via phone calls or face-to-face interactions with providers who participated in the focus group discussions. The interviews took place in a private space that was selected by the interviewee—no one else was present except for the interviewer and interviewee. The topic guide included questions focused on individual family planning choices for the respondents, as well as broader questions about the strengths and weaknesses of Rwanda’s family planning program related to the nation and the community, including the topic of unmarried adolescents.

Male and female Rwandan university undergraduate students conducted the focus group discussions and in-depth interviews. Training included instruction on qualitative methods, focus group discussion and interviewing techniques, ethical research, and practice with the topic guides. Included in the training were additional instruction on the sensitive nature of the topics and techniques the data collectors could use to put the respondents at ease. Trained data collectors pilot tested the topic guides in practice with each other. The study participants and data collectors did not know each other prior to data collection. They met just before data collection, exchanged pleasantries, and shared the study objectives via review of the consent form. Each individual signed a consent form prior to data collection. The consent forms included the purpose of the study, how the results would be used, what the data collection would entail, the anticipated duration of participation, anticipated risks and benefits, the voluntary nature of participation—overall and within the study, the anonymous storing of the data and sharing of the results, as well contact information for further questions or concerns. Potential study participants were offered to read, or be read, the consent form based on their needs and preferences. The focus group discussions and in-depth interviews were all conducted in Kinyarwanda and audio recorded with study participant permissions. There were no participants who refused to participate nor dropped out after consenting.

Audio recordings and written notes were translated at the field sites into English by the authors, including native Kinyarwanda and English speakers, and transcribed verbatim. The transcripts were not shared with study participants. Analysis was guided by thematic content analysis. Transcripts were coded in Atlas.ti version 8. Two teams of 10 research team members coded the transcripts. Each team of 10 coded 1 transcript together, then shared the results of coding in terms of code names, and a discussion ensued to develop a common code dictionary. Then each team member coded the remaining transcripts, and results were compared during analysis of each code. Themes were drawn from the data, not defined a priori. Codes were then further analyzed using excel spreadsheets, with participants in the rows, codes in the columns, and quotes in the cells.

Institutional Review Board approval was obtained at Western Washington University as well as with the Rwandan Ministry of Education prior to data collection. Study participants received 10,000 Rwandan francs (~ $11USD) for their time and transport costs.

## Results

To contextualize the results, the following is a summary of the demographics of the sample. Half of the IDI participants resided in Musanze, while a bit more than half of the nurses and CHWs did as well. The average age of IDI participants was 38, compared to 40 among nurses and 47 for CHWs. IDI participants had on average 4 children while nurses had 3 and CHWs had 5. All IDI participants were female compared to 95% of the nurses and 54% of the CHWs. The most common method used by the CHWs (42%) and IDI participants (41%) was injectables, while it was the implant (55%) for the nurses (see Table 1).

**Table 1:** Sociodemographic characteristics of family planning providers and experienced contraceptive users, Rwanda 2019

	FP nurse (n = 40)	CHW (n = 48)	Experienced user (n = 32)
Districts, % Musanze	53	52	50
Age, mean (SD)	40.3 (6.7)	46.8 (7.1)	37.7 (6.0)
Parity, mean (SD)	2.9 (1.3)	4.6 (1.5)	3.9 (1.8)
Sex, % female	95	54	100
Contraception used, %	90	90	100
Condom	3	15	22
Pill	0	21	13
Injectable	18	42	41
Implant	55	17	19
IUD	13	0	0
Sterilzation	8	17	4

Three major themes arose from the analysis: stigma about premarital sex creates access barriers for youth, family planning providers both restrict and facilitate access to adolescents, and adult modern contraceptive users are mixed on communicating with their children about family planning but want youth to have access to contraception.

### Stigma about premarital sex creates access barriers for youth

Within the theme, stigma about premarital sex creates access barriers for youth, two subthemes emerged: contraceptives are for married people as well as adolescents feel shame when accessing family planning services.

#### Contraceptives are for married people

All study participants noted the difficulties adolescents face in accessing contraceptives. The stigma surrounding adolescent contraceptive use stemmed from the cultural norm that sexual activity is confined to marriage; therefore, unmarried youth should not need to access contraceptive services.

…where I work the people we give service to are people who are married.

Nurse, female, Nyamasheke

I would advise her that, at the time she is married and living with a partner, then she may use contraceptives.

Condom user, Nyamasheke

Some felt that providing services was akin to encouraging adolescents, and by extension as well as their peers, to engage in sexual activity.

…I think that if a young girl went to use the contraceptive program it would be a problem. Using contraceptives is important, but for me I have a girl, and I can’t advise her to use contraceptives because it will permit her to have sex…

Condom user, Nyamasheke

…I will keep her secret and not tell anyone else the same age that she is using family planning because they think that we give them the freedom to have sex anytime and any day.

CHW, female, Musanze

#### Adolescents feel shame when accessing services

Participants explained that when youth do access contraceptive services, it is assumed that they are selling sex for money.

We have youth who come to us, for example…where I work…most of the youth who come to me are prostitutes…

CHW, male, Musanze

…they think that people will laugh at them because they are going to use contraceptives. They laugh at them because they think that if an adolescent is using contraceptives then she is a prostitute.

Injectable user, Musanze

Due to the cultural taboo around sex before marriage, reaching out to access contraceptive services from family planning providers for unmarried youth is associated with shame and fear, therefore, youth avoid doing so.

…some young ladies are afraid to go to the health center because she thinks that if somebody sees her where they give the service of family planning, this will be shameful.

CHW, male, Musanze

…she is still young and has done terrible things...in our culture, as young people it is not simple to get information about family planning and to see the person who can give you contraceptives…She is ashamed to go to the health center to seek information because people can see her and she is not married.

Nurse, female, Musanze

Youth use strategies to access services in ways that conceal their identity.

…youths, they are coming in hiding.

Nurse, female, Nyamasheke

She may come and hide and talk to the doctor, “I want to use a contraceptive method, but I don’t want my family or sister to know I came here for family planning service.”

Nurse, female, Nyamasheke

One strategy employed is using services further from their homes to avoid running into neighbors.

…people don’t come to us because they are afraid, but they go to another health center that is far away so that people won’t know them.

CHW, female, Musanze

Another strategy is to access services anonymously.

That’s why I think that it’s only someone who has courage who will come and ask you directly about family planning…They might ask someone for your number to call you, but they don’t want you to know that it’s her who is calling.

CHW, male, Musanze

Study participants blamed adolescents for not accessing and using family planning services.

But for me, I think that girls also need to use contraceptives. But the adolescents don’t see that it is acceptable for them to use contraceptives.

Injectable user, Musanze

The government should educate the youth to not have sex without using condoms or without having a contraceptive method. But, I think that the government does do that and gives this information to the youth, but the youth doesn’t understand it still because they don’t believe the information as much. But we will continue to teach and show them the danger of unprotected sex without using contraceptives or condoms and show them that the consequences don’t only affect your parents, but will also affect you and your whole country.

Pill user, Musanze

### Family planning providers both restrict and facilitate services for adolescents

Within the theme, family planning providers both restrict and facilitate services for adolescents, there are six subthemes: providers are caring with youth; providers stress secondary abstinence with adolescents; youth need private, confidential, and efficient services; limited contraceptive selection offered; emphasizing risks; and youth corners. Providers emphasized facilitation of service provision to youth more than barriers.

#### Providers are caring with youth

Most providers indicated they would provide services to adolescents. Providers admired adolescents’ decisions to avoid pregnancy, and expressed thanks to unmarried women who sought family planning as an alternative to pregnancy.

…I have met with different girls who come to me first for advice. After they ask me for advice, I tell them they should go to the health center because it is better to use a contraceptive method than to get pregnant.

CHW, female, Musanze

Providers noted the care they would take with youth—how they would welcome them, make them comfortable, and treat them with kindness and respect.

The important thing is that we welcome her and behave well in front of her because when she comes to see you and tells you that she wants to use family planning, you should not look surprised or angry, but welcoming instead. Because if you look upset, she will think that her request is a big problem. So we are prepared on how to behave in that sort of situation. Treat her like your own daughter. You have to flatter her and let her know that she did the right thing for coming to see you. She is courageous because she does not care what people think about her.

CHW, male, Musanze

First of all they will receive her by thanking her for her decision it took for not wanting to become pregnant, because it’s not normal that…young adults will want to use family planning. So they will thank her and give her advice on methods.

CHW, female, Nyamasheke

#### Providers stress secondary abstinence with adolescents

While most providers indicated they would provide contraceptive services to adolescents, they also noted how they would emphasize secondary abstinence, using their friendly demeanor and verbal persuasion to convince youth to stop having sex.

After having friendship with this youth, that way you might be able to help her leave what she is doing because the first thing you showed her was that there isn’t a problem, you showed her friendship. After building a friendship, that’s when you might be able to help her by telling her what she’s doing is wrong, can you reduce this or that, because after she’s able to trust you we’ll be able to help her...

Nurse, female, Nyamasheke

If she does not agree to stop having sex, the CHW can advise her to use a contraceptive method in order to prevent unwanted pregnancy.

CHW, male, Nyamasheke

…the CHW will explain the good side of not seeing her boyfriend so she doesn’t get pregnant but will also tell her about using protection… She can continue her business but without bringing more kids into the world.

CHW, male, Musanze

#### Youth need private, confidential, and efficient services

Family planning providers emphasized the need to receive youth privately as to avoid others knowing their desire to use contraception.

She will be well received. She, who doesn’t have a husband… we have a certain way to receive her, because we want to protect her privacy. We will receive her in a private place. We don’t want everyone to see that she is here for family planning.

Nurse, female, Nyamasheke

…there is a time when they come to the health center to get family planning but it is done in a secret way that other women can’t know that they use family planning.

Implant user, Nyamasheke

At the health center they must receive her in strong secret in order for her to feel like she can give more information as it is custom for those who have husbands to get family planning services.

CHW, female, Nyamasheke

…when they come and meet at my house, you can tell her to come back when others are not there and teach her in secret…

CHW, female, Musanze

Family planning providers also noted the importance of maintaining confidentiality when working with youth.

…contraception is not used by women or men only. There are even youth who use them. They have challenges of coming to see a CHW…and in that time you have to comfort them….you will take them to the health center and they will help them and you make sure it stays a secret between the doctor, CHW, and person who needs to use contraception.

CHW, male, Musanze

I have a girl who came to me and told me that she is having sex with her boyfriend. The first thing I did is I guaranteed her that I will keep it a secret and help her to get contraceptives. So I had a discussion with her and told her how to proceed. I had to keep her secret.

CHW, female, Musanze

CHWs were noted as safer providers for youth—mostly so youth can avoid the shame of accessing services publicly where married persons go for services. When youth needed to go to the health center, CHWs helped youth interact with providers at the health center, privately.

She must first go to the Community Health Workers where she can get a method like condoms. She may continue having sex with her boyfriend- it’s easier to go through the Community Health Worker because she might feel free instead of going to a clinic.

CHW, male, Nyamasheke

Since she is not pregnant, unmarried and a youth, she cannot go seek out contraception at the health center alone. So the CHW will speak to those family providers in confidence in order to help her receive contraception.

CHW, female, Nyamasheke

While others suggested nurses were the better option for youth due to confidentiality reasons.

…youth can’t go to community health workers because…a community health worker can break my secret and tell someone that I am using contraceptive methods and I don’t have a husband and that’s why women prefer to go to the health center to talk to the nurses because the nurses cannot break the secret.

Sterilized, Nyamasheke

If youth do come for services, they plead for urgent services to avoid being seen by others.

…they are seen very fast. Some people might say since she’s using family planning, she wants to continue having sex. She has to be seen very fast.

Nurse, female, Nyamasheke

The youth want to come without being seen by the married women. If they come, they say, “Please, doctor, please do me a favor and give me fast service so those people won’t see me.” As a nurse that’s a challenge because we don’t have enough time to discuss all the contraceptive methods they can choose from. They want to leave so fast so no married women can see them searching for contraceptive methods.

Nurse, female, Musanze

#### Limited contraception selection offered

The contraceptive methods given to youth were more limited than the universe of possible contraceptive options available to married women.

The first thing that the doctor will do is to be confidential and refer her to the youth corner service and explain to her again the methods that you offer for adolescent people.

Nurse, female, Nyamasheke

The limited selection provided to adolescents was linked to fears of infertility at later stages, when the youth was married and desiring pregnancy.

…we have to choose for her a good method that will not prevent her from getting pregnant if she wants to get pregnant at the time she gets married.

Nurse, female, Nyamasheke

Family planning providers most often gave youth condoms and pills.

…I will give her advice “even though you have had sex at a young age, the ways of using contraception is like this…” I would give her examples and let her choose which one will work for her. But first I will teach her to stop having sex, if we see that she can’t then I will just give her condoms.

Nurse, female, Musanze

The young girls…They tell me that they use pills…they tell me that it’s the best method that the nurses choose for them, because it will not cause side effect for them.

Condom user, Nyamasheke

For those who are young, the teaching method is different for family planning because I am not going to have a 14-year-old come to me and I will teach her about the five-year implant, because the older you are the more education you get. For young person, I will teach her about calendar method and condoms.

CHW, male, Musanze

Only one study participant, who was not a family planning provider, advocated for longer term method use among youth.

…I think that the youth for now the nurses need to get closer to them and give them five-year implants, I think this because in today’s society the youth does not behave well. I think that nurses should pay more attention to the youth so that the youth can avoid unwanted pregnancy.

Condom user, Nyamasheke

#### Emphasizing risks

Providers emphasized potential risks with contraceptive use when advising adolescents, more so than with married women.

…you have to show her that she can’t keep having sex because even a family planning method can fail and she can still get pregnant using contraception. You have to show her all the negatives so she can know the things to do to take care and protect herself.

CHW, female, Musanze

While the topic of human immunodeficiency virus (HIV) never arose when providers were discussing their interactions with married women, it was a common concern when advising adolescents.

…a CHW will welcome her just like how she will welcome another women who will come to her for help in the village without abandoning her. You see there are a lot of girls that you know in the village who are having sex, you can’t abandon them instead you have to welcome them like a parent after you meet you can tell her the bad side about having sex without using protection because she can get sexually transmitted diseases.

CHW, female, Musanze

…when those who are not married come to ask for family planning, this makes us happy, because we help them prevent unwanted pregnancy. If she is courageous we will teach her about family planning deeply as my colleague said. We will teach her about all family planning methods until we help her to choose the best family planning method that will help her, but we also tell her that because she is not married and is having sex, she can contract HIV…

Nurse, female, Nyamasheke

Additionally, for pregnant youth, exposure to pregnancy during the postpartum period was treated more urgently than for married women.

…the CHW will not consider the doctor’s appointment of the 6-month plan after giving birth. I will go tell the doctor she is pregnant and wants to use family planning. After giving birth the doctor will give her contraception…He will find a method before sending her home, because she can get pregnant in those 6 months after birth.

CHW, male, Musanze

…if she gives birth the doctor who takes care of her should give her a method of contraception right at that time because she may not want to come back again because she may be ashamed to come back seeking a contraceptive when she is still not married. It would be good if she got a contraceptive method after giving birth.

Nurse, female, Musanze

#### Youth corners

Youth corners, spaces in health facilities designed to be safe places for youth to access reproductive health services, were presented as a panacea for youth.

She is most welcome to the youth corner, because in the youth corner we take care of youth, both young and underage, so we can teach them about reproductive health. In the youth corner they meet only the young people so they can be comfortable with discussing with each other. They have some games to play so they can be comfortable with each other. The youth corner is located via the playgrounds so they can come for playing and a doctor can teach them about they can get pregnant if they have unprotected sex. There is no shame on her for going to the youth corner.

Nurse, female, Musanze

If she goes to the health center without using the ‘Youth Corner’. The provider may ask her why she did not come to the center with her husband. They may consult with her village CHW to find out more about her specific case. She may not be received well at the health center; however, if she goes to the ‘Youth Corner’ she will be received without judgment.

Nurse, female, Musanze

…in that corner she is able to freely express and go directly to the person she needs and doesn’t have to run into her mom, peers, or neighbor.

Nurse, female, Musanze

### Adult contraceptive users are mixed on communicating with their children about family planning but want youth to have access to contraception

Within the theme of adult contraceptive users are mixed on communicating with their children about family planning but want youth to have access to contraception there are two subthemes: communicating with children about family planning and wanting youth to have access to contraceptives.

#### Communicating with children about family planning

Modern contraceptive users were asked about their intentions to or experience with conversing with their own children about family planning. Some women indicated comfort with these conversations, and nearly the same amount indicated discomfort.

I: How do you feel about talking about these things with your kids?

R: When I talk about these things with sex and family planning, the kids become embarrassed and it makes me also uncomfortable in front of my children. Sometimes they laugh and I tell them to laugh but it’s advice that I’m giving them. It’s not something big. I have to take care of my kids and give them the necessary advice.

Injectable user, Musanze

I: Do you take time to discuss family planning with your children?

P: No, it is hard to take time to talk about sex and how to use condoms with my kids. It is embarrassing.

Injectable user, Musanze

Among those who did talk about sex with their children, most indicated it was difficult in the beginning but the challenges decreased with time and experience.

I: Were you comfortable when you first started having these conversations with your children?

P: The first time it was hard time but now it is a normal discussion.

Condom user, Nyamasheke

#### Wanting youth to have access to contraceptives

Most modern contraceptive users want youth to have access to contraceptives—to avoid unwanted pregnancies, which are viewed as a burden to young girls as well as for the entire community.

…I think that also girls (unmarried women) need contraceptives because they are the ones who have problems right now, they have problems of getting unwanted pregnancies…

Condom user, Musanze

…for girls, you can’t protect them from the diseases that are outside, but you can protect them from having unwanted pregnancies. But if she uses contraceptives, it can protect her from unwanted pregnancies and reduce the problem in the village of young women having to say which man got her pregnant…This will help us to improve ourselves and give us a better future, because there will be no babies that are a burden to our daily life in the village.

Pill user, Musanze

Perhaps because they could directly relate to the potential consequences of unplanned pregnancies out of wedlock, modern contraceptives users were on average far more accepting of youth using contraception than providers.

## Discussion

This study aimed to explore Rwandan family planning provider and female adult modern contraceptive user perspectives on adolescent contraceptive use. Results demonstrate that stigma regarding premarital sexual activity limits contraceptive access for adolescents, and that while providers will meet with adolescents, services are provided with barriers. The study also found that adult women in the communities who use modern contraception themselves recognize the importance of and approve of youth access to family planning services. Despite sampling by district purposively, there were more similarities than differences in findings between the two districts.

Participant’s comments about adolescent access to contraception demonstrate that stigma regarding premarital sexual activity is persistent. In Rwandan culture, sex only occurs during marriage; therefore, seeing an unmarried youth in a space for married adults violates those norms, a social standard present in other cultures as well [8, 15, 31–34]. As a result, youth feel shame and fear in accessing family planning services and as a result largely avoid accessing services. Youth who do access services were labeled prostitutes. Study participants worried that provision of contraception to youth would encourage them to engage in more sexual activity, a fear noted in other settings as well [15]. Simultaneously, within this context, youth were blamed for not accessing contraceptive services in cases of adolescent pregnancy due to not understanding the importance of contraception. Research among providers in South Africa also reported blame of low adolescent service utilization shift back onto adolescents [11]. National and community level sensitizing efforts are necessary to reduce stigma and increase youth access to services [8, 35]. Given that the stigma regarding premarital sexual activity is resulting in barriers to youth accessing contraceptive services, it is imperative governments and family planning programs work to destigmatize adolescent use of contraception in an inclusive and intersectional manner [16, 27, 36].

While positive contributions regarding serving adolescent clients were more common, providers also noted ways they would restrict adolescent access to services. Providers were eager to emphasize the risks of sexual activity, even with contraceptive protection, such as contraceptive failure, sexually transmitted infection (STI) exposure, and no birth spacing. While the possibility of contraceptive failure is an important conversation with any client—it was concerning that this topic only arose when providers discussed counseling adolescent clients. This is particular problematic given that contraceptive failure is more common among adolescent contraceptive users compared to adult contraceptive users [37]. Similarly, STI prevention is an important topic around any counseling regarding sexual activity and risky health behavior; however, providers only mentioned STI risk without discussing risk mitigation efforts, such as condom use, when sharing how they counseled youth. Again, this topic did not arise in discussions of counseling adult women. The emphasis placed on postpartum contraceptive uptake with youth is important, yet, as with the other points noted here, should be emphasized with all clients—not just youth.

Providers also discussed the persuasive techniques they used to convince youth to stop sexual activity and engage in secondary abstinence. In other settings, research has shown that most providers encourage secondary abstinence, as well [38]. Studies have found, however, that it is easier to change contraceptive behavior as compared to sexual behavior [6]. A key difference in this study is that providers did offer contraception if their efforts to convince youth to abstain failed [15].

Additionally, providers reported narrow method selection for youth focusing primarily on pills and condom provision, which stems from a concern that using long-term methods of contraception will negatively impact fertility, a fear common in sub-Saharan Africa nations [13, 34]. Researchers have noted that condoms are a good choice for adolescents given their infrequent sexual activity [39]; however, long-acting methods that are more effective and provide protection for longer periods of time can also be promoted with adolescents [36]. Studies indicate how pervasive provider biases regarding age are—and how they increase with method effectiveness [40]. Survey data show that adolescents in Rwanda are increasingly opting for implants and injectables over pills and condoms. Providers emphasized HIV risk with adolescents and offered condoms to adolescents, without also noting the role of condoms in dual protection for these young clients. Governments and family planning programs must reduce stigma toward adolescent sexual activity among family planning providers, as adolescents often access contraception from public services [41]. Family planning providers, and all staff who interact with adolescent clients, should receive additional training to provide services to meet their clients contraceptive needs, regardless of their age or marital status [8, 10, 15, 42, 43].

Family planning providers described the care they would take when receiving and counseling youth clients, opposing findings in Uganda [13], but shared in Ghana [31]. Providers were particularly sensitive to seeing youth in private, maintaining their confidentiality, and providing expedient services—areas raised by providers and youth as concerns with service access [8, 11, 13, 16, 42, 44]. Providers described youth corners—specific spaces within some health centers designed and staffed specifically for youth contraceptive service provision—as a welcoming place for youth to access services. Family planning providers in Rwanda feel that the youth corners are the key to youth access. While researchers deemed it unnecessary and impractical, family planning providers in South Africa requested youth designated spaces in their facilities [42]. Future research should explore the impact of youth specific spaces on youth access from the perspective of youth, given that youth don’t prioritize these spaces [10]—as well as the impact of youth specific spaces on contraceptive use.

Due to limited funding and space constraints, not all health facilities in Rwanda have youth corners, a common limitation in other settings as well [42]. Research on youth centers in other settings shows minimal impact [45]. Youth corners share some qualities with health posts, another Rwandan government workaround to contraceptive service barriers. Health posts are stand-alone centers near health centers affiliated with the Catholic Church—in order to make contraceptive access available to those who fall within Catholic health facility catchment areas, as the Catholic-run health facilities refused to include modern contraceptive methods in their offerings.

Another important finding is that adult female modern contraceptive users were more open to youth access to contraception than providers, possibly due to feeling more acutely the consequences of adolescent unwanted pregnancy within their communities. Research on adolescent access to and use of family planning often notes barriers at the community level—without actually including community members in the study. This study included community members and found less stigma about premarital contraceptive use than was found among providers. This suggests that cultural “norms” may in fact not be as universal as previously considered. Community members understand the delicate balance between adherence to community standards with the lived realities of sexually active unmarried youth. These findings suggest that future research on adolescent access to contraception should include community members and to allow for openness to contraceptive access for youth. Creating spaces for dialogue between family planning providers and community members about adolescent access to contraception services might also contribute to reducing the stigma toward premarital sexual activity and adolescent access to services—and subsequently allow providers the space to question their own stigma and biases.

The modern contraceptive users were mixed in their comfort in engaging in conversations about contraception with their own youth. Given the importance of trusted adults for youth on any topic, and in particular topics around sex, providing more parents and caregivers with the tools and support to talk with their youth about sex and contraception will likely contribute to reductions in unsafe behaviors for youth [38, 46–48].

This study has a number of strengths. Inclusion of three different study populations: family planning nurses, community health workers, and adult modern contraceptive users allowed for triangulation of data, and important insights into the convergence as well as divergence of findings. In particular, the inclusion of adult modern contraceptive users allowed for a unique perspective often left out of research on adolescent contraceptive access and use. Data were collected from two different districts, purposively selected for having the highest and lowest contraceptive prevalence rates in Rwanda.

Despite several strengths, some limitations must be noted. Primarily, adolescents were not included in the study, leaving absent the adolescent experience and perspectives. Future research on adolescent contraceptive access in Rwanda will benefit from inclusion of this vital perspective. Additionally, translation and transcription occurred in a single step, decreasing the accuracy of translation. Finally, providers recruited experienced contraceptive users, which likely led to recruitment of participants that had more positive experiences with contraceptive service provision and use.

## Conclusion

This study shows that both family planning users and providers observe that there is a need for greater access to contraceptive services for youth; however, family planning providers and community members highlight the needs and challenges for adolescents seeking family planning services prior to marriage. Removing stigma around youth access to contraceptive services will alleviate significant barriers adolescents currently face. Making the behavior normative will reduce stigma, and one way to initiate that is to motivate people to talk about the subject interpersonally with family and social networks, within the community, and nationally. Governments should engage providers in discussions as well. This can occur through training, but the training must be nuanced in order to address the personal biases of the providers, which stem from the broader cultural taboo of premarital sexual activity. The current solution of increasing youth access to contraception via youth specific spaces may work as an interim solution; however, in the long-term youth access to contraception requires nationwide reduction in stigma toward safe and informed premarital sexual activity.

## Data Availability

The data generated and analyzed during the current study are available from the corresponding author on reasonable request.
